# Substitution of Animal Fat and Sodium Nitrite with Hemp Seed Oil: Effect on the Nutritional Value, Sensory Characteristics, and Shelf Life of Fermented Salami

**DOI:** 10.3390/foods13162584

**Published:** 2024-08-18

**Authors:** Georgios Papatzimos, Zoitsa Basdagianni, Eleni Kasapidou

**Affiliations:** 1 Department of Agriculture, University of Western Macedonia, Terma Kontopoulou, 53100 Florina, Greece; dagro00004@uowm.gr; 2 School of Agriculture, Department of Animal Production, Aristotle University of Thessaloniki, 54124 Thessaloniki, Greece; basdagianni@agro.auth.gr

**Keywords:** fermented salami, hemp seed oil, sodium nitrite, proximate composition, microbiology, fatty acid profile, colour, texture, sensory traits

## Abstract

Recently, products of plant origin have been utilized to extend the shelf life of meat products. This study examined the impact of hemp seed oil as a replacement for animal fat and sodium nitrite on the nutritional, physicochemical, technological, and sensory traits of fermented salamis. Five treatments were prepared: S0 (100 mg/kg NaNO_2_), S1 (2% hemp oil and 50 mg/kg NaNO_2_), S2 (4% hemp oil and 50 mg/kg NaNO_2_), S3 (2% hemp oil), and S4 (4% hemp oil). The addition of hemp seed oil did not affect proximate composition but improved fatty acid composition and lipid quality nutritional indices. Microbial growth was consistent across all treatments. Active acidity (pH) and water activity (aw) were influenced by hemp seed oil and/or sodium nitrite. Salamis containing only hemp seed oil exhibited lower redness and chroma values during storage. Hemp seed oil led to higher lipid peroxidation, mitigated by sodium nitrite. The addition of hemp seed oil and varying levels of sodium nitrite significantly impacted salami texture. Sensory evaluation showed consumer acceptance of hemp seed oil-enhanced salamis. In conclusion, hemp seed oil can be used as a functional ingredient to improve the nutritional value and healthiness of fermented meat products when combined with reduced sodium nitrite content.

## 1. Introduction

Meat fermentation is a traditional preservation technique commonly employed to enhance the quality and extend the shelf life of the products. These items are highly popular among consumers, and currently, about 20–40% of all processed meat products in European countries are fermented, with sausages comprising the majority. Common examples of fermented sausages include salami, mortadella, genoa, pepperoni, and cervelat [1,2]. According to the definition by Ordonez et al. [3], dry-fermented sausages are meat products that involve the selection, chopping, and mincing of meat and fat (with or without offal), along with the addition of condiments, spices, and additives. This is followed by a process of ripening, curing, and occasionally smoking.

However, lately, there are growing concerns over the health aspects of meat products, with consumers demanding healthier options that are low in salt, fat, saturated fatty acids, cholesterol, and calories. Furthermore, there is pressure for the production of low-nitrite or nitrite-free products due to the fact that sodium nitrite in meat products is linked to the potential formation of carcinogenic N-nitrosamines [4,5,6,7]. Nitrite is frequently used in fermented meat products due to its antimicrobial properties, especially against *Clostridium botulinum*, its capacity to prevent oxidation, and its contribution to the distinctive cured colour and flavour [6]. Plant-derived additives are being explored by the meat industry to reduce or eliminate synthetic ingredients, as there is a common belief that natural compounds are safer, despite the regulated safe use of nitrites within set limits [8].

Product reformulation is a relative new strategy to develop products with improved nutritional value while ensuring the willingness of the consumers to pay a premium price for their demands [9]. In this respect, vegetable oils can help reduce unhealthy components, such as saturated fats, while also introducing beneficial bioactive compounds like polyphenols, renowned for their antioxidant and antimicrobial properties [10,11,12]. The meat industry is innovating by reducing the fat content and additives such as nitrites or by substituting animal fat and synthetic nitrites with vegetable fat and alternative “green” nitrites, respectively.

Replacement of animal fat with plant-based oils can be achieved either by direct inclusion of these oils or pre-emulsified oils. Canola oil, olive oil, linseed oil, soyabean oil and chia oil have been used to substitute animal fat (pork, lamb and beef) in various meat products such as bologna, frankfurter, pâtés, fermented sausages as well as burgers and patties, as reported in recent review studies [13,14,15]. Incorporation of vegetable fat affected characteristics such as colour, texture, flavour, oxidative stability but the effect is variable based on the type of vegetable oil, the type of meat product, the substitution percentage, etc. [13,16].

Nowadays and following hemp legalization, hemp seed oil can also be used in the formulation of meat products. Montowska et al. [17] reported that addition of hemp seed oil in pork meatballs reduced the content of saturated fatty acids and the extent of protein and lipid oxidation during storage. Botella Martínez et al. [18] found that either total or partial replacement of fat by a gelled emulsion consisting of hemp oil and buckwheat flour did not affect the technological and sensory characteristics of frankfurters. In a similar study, the use of amaranth flour hemp oil gelled emulsion as pork backfat substitute improved the nutritional properties without affecting technological or sensory traits of beef burgers [19]. Finally, Augustyńska-Prejsnar et al. [20] found consumer acceptance of poultry roast is dependent on enrichment level with both hemp flour and hemp oil where products with a higher oil content received lower scores in relation to taste and binding. The effect of application of hemp seed oil in meat products has not extensively explored in relation to the extensive studies conducted in other plant—based oils [13,16].

Hemp seed oil is a major component of the *Cannabis sativa* L. plant, comprising about 35% of the seed. The health and nutritional advantages of hemp seed oil result from its abundant polyunsaturated fatty acid (PUFA) content [21] and the presence of additional beneficial minor components like tocopherols and polyphenols [22].These compounds possess strong antioxidant properties protecting the oil from oxidation and offering health benefits to humans [23]. The European Union has sanctioned the cultivation of hemp varieties with up to 0.2% THC (tetrahydrocannabinol), a psychoactive compound [24]. Hemp is used in essential oils, food, personal care products and medical preparations and it is generally estimated that the use of industrial hemp will substantially increase [25]. Hemp-based foods are marketed as offering various health benefits. Consequently, there is a promising growth trend in producing novel foods from industrial hemp [26]. Considering the above, the meat industry could use hemp products as an alternative ingredient in processed meat products to enhance their quality, especially regarding the fatty acid profile and the content of natural preservatives. This trial aimed to study the effect of hemp seed oil addition in relation to partial or complete replacement of sodium nitrite in the nutritional value and shelf life of fermented salamis.

## 2. Materials and Methods

### 2.1. Product Preparation and Treatments

Before sample preparation, the ingredients were initially weighed and stored at the appropriate temperature until use. Specifically, pork and beef were trimmed of visible fat and stored in the freezer (−18 °C) for 2–3 days. They were then thawed in the refrigerator (2–4 °C) until the production process began. Pork backfat, stripped of adhering skin, was also stored in the freezer and left at room temperature one hour before cutting. BactoFlavor^®^, BFL-T03 (Chr. Hansen GmbH, Pohlheim, Germany), served as the starter culture. This lyophilized fermentation product was stored at −18 °C until use and hydrated with water immediately before application for activation. The proportions of the ingredients for each treatment are presented in Table 1.

**Figure 1:**
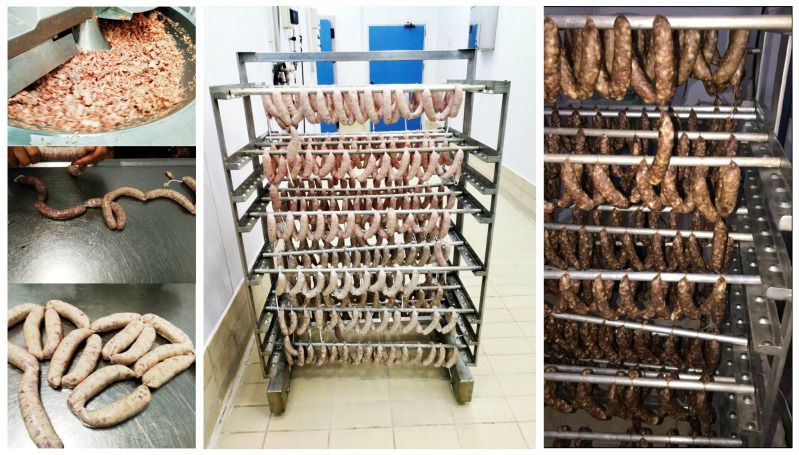
Salami production procedure.

Salamis were placed in the ripening chamber for 18 days (Figure 1), following conditions applied by the meat industry as shown in Table 2. At the end of the ripening period, the samples were vacuum-packaged, with each package containing four salamis, and preserved at −2 °C to +2 °C for 90 days in refrigerated display cabinets to simulate retail conditions.

### 2.2. Sample Collection and Preparation for Chemical Analyses

Sample preparation for chemical analyses was conducted as described in Kasapidou et al. [27]. Briefly, analyses were performed on samples of approximately 200 g each, consisting of 3–4 salamis. The samples were finely ground in a domestic food chopper to ensure uniformity. The homogenized samples, intended for proximate composition determination, were stored in airtight plastic containers to prevent moisture loss and minimize air gaps, avoiding deterioration during storage at 4 °C. Before analyzing the chemical composition, the samples were meticulously blended using a spatula by hand. All analyses were completed within one week of sample collection. Specimens for the analysis of fatty acid composition were vacuum-packaged and stored at −20 °C until analyzed. Samples for the of lipid oxidation were prepared on the day of analysis. Each analysis was carried out twice for accuracy.

### 2.3. Proximate Composition and Residual Nitrite Analysis

The proximate composition of the salami samples was determined using AOAC standard methods [28], as detailed in Papatzimos et al. [8]. The moisture content was determined using method 950.46, which includes drying the sample in a convection chamber (102 °C) until a consistent weight was achieved. Ash content was determined using the method 920.153, with samples incinerated (550 °C; 12 h) until light grey colour ash was obtained. Protein content was determined by the Kjeldahl method 928.08, with nitrogen content converted to crude protein by multiplication by 6.25. Fat content was assessed using the Soxhlet 991.36 method through extraction with petroleum ether. The content of sodium chloride was determined by a modification of the 937.09 Volhard method, involving excess silver nitrate addition followed by back-titration with a standard ammonium thiocyanate solution, using saturated ferric ion as an indicator. Residual sodium nitrite was determined following the International Standard-ISO 2918(E) method [29], where nitrites were extracted from the sample, and their concentration, expressed in mg/kg, was calculated using a calibration curve.

### 2.4. Fatty Acid Composition and Nutritional Indices

Samples were thawed overnight at 4 °C and the next day fatty acids were extracted and methylated following the method of O’Fallon et al. [30] as described in Papatzimos et al. [8]. The sample was placed in a screw-capped Pyrex tube with aqueous KOH solution and methanol. The tubes were then placed in a water bath at 55 °C for 90 min and shaken vigorously by hand for 5 s every 20 min to ensure proper permeation, dissolution, and hydrolysis of the sample. Following cooling in an ice water bath, aqueous H_2_SO_4_ was added, and the tube contents were gently mixed by inversion. Then the tubes were returned to the water bath (55 °C; 90 min) with intermittent shaking. After cooling again in an ice water bath, hexane was added, and the tubes were vortexed for 5 min before centrifugation at 1100× *g* for 10 min. The upper phase was filtered through a PVDF syringe filter (0.45 µm pore size), transferred into amber GC vials, and stored (−20 °C) until analysis.

The analysis of fatty acid methyl ester was performed using an Agilent Technologies 6890 N gas chromatograph equipped with a flame ionization detector (FID) and a DB-23 capillary column (60 m × 0.25 mm i.d., 0.25 μm film thickness, Model Number: Agilent 122 2362). The injector temperature was set at 250 °C, and the GC operated with helium as the carrier gas in split mode injection (50:1, 3 μL). Injector and flame ionization detector temperatures were maintained at 250 °C and 300 °C, respectively. The analysis started with an initial oven temperature of 110 °C for 6 min, followed by a gradual increase to 165 °C at 11 °C per min, then to 195 °C at 15 °C per min, and further to 230 °C at 7 °C per min, with a 7-min hold at 230 °C. Identification of fatty acids was performed using three different commercial standard mixtures, including a 37-component FAME mix, PUFA-2 (Animal source), and a blend of cis- and trans-9,11- and -10,12-octadecadienoic acid methyl esters as reference standards. Quantification was based on peak area measurement, with results expressed as percentages (%) of the total peak areas for all quantified acids. Fatty acids were categorized into saturated fatty acids (SFA), unsaturated fatty acids (UFA), monounsaturated fatty acids (MUFA), and polyunsaturated fatty acids (PUFA).

The fatty acid profile was employed to assess the following nutritional indices related to healthy fat consumption, as specified in the review by Chen and Liu [31], for meat and meat products. Their review systematically gathered data on fatty acid composition from studies published since 2000 to deepen comprehension of various nutritional indices’ implications and applications.

Polyunsaturated fatty acid/Saturated fatty acid ratio

$$PUFA/SFA=\frac{\Sigma PUFA}{\Sigma SFA}$$

Atherogenicity Index

$$AI=\frac{\left(C12:0+(4\times C14:0)+C16:0\right)}{\Sigma UFA}$$

Thrombogenicity Index

$$TI=\frac{C14:0+C16:0+C18:0}{((0.5\times \Sigma MUFA)+(0.5\times \Sigma n-6PUFA)+(3\times \Sigma n-3PUFA)+(n-3PUFA/n-6PUFA))}$$

Hypocholesterolaemic: hypercholesterolaemic fatty acid ratio (h/H)

$$h/H=\frac{C18:1n-9cis+\Sigma PUFA}{\left(C12:0+C14:0+C16:0\right)}$$

### 2.5. Microbiological Analyses

Microbiological analyses were conducted on storage days 30, 60 and 90. International Organization for Standardization (ISO) methods were used for the enumeration/detection of microorganisms: ISO 4833-1, 2013 [32] for total aerobic microorganisms; ISO 21527-2, 2008 [33] for yeasts and moulds; ISO 15214, 1998 [34] for mesophilic lactic acid bacteria (LAB); ISO 21528-2, 2017 [35] for Enterobacteriaceae; ISO 4832, 2006 [36] for coliforms; ISO 16649-2, 2001 [37] for β-glucuronidase-positive *Escherichia coli*; ISO 6888-1, 2021 [38] for *Staphylococcus aureus*; ISO 6579-1, 2017 [39] for *Salmonella* spp. and ISO 11290-1, 2017 [40] for *Listeria monocytogenes*.

For total viable aerobic mesophilic microorganisms, 25 g of the sample was homogenized in 225 mL of buffered peptone water, diluted, plated on Plate Count Agar, incubated at 30 °C for 72 h. Yeasts, moulds, and fungi were similarly prepared, diluted, and plated on Dichloran Rose-Bengal Chloramphenicol agar, incubated at 25 °C for 5 days. Mesophilic lactic acid bacteria were diluted in MRS broth, plated on MRS agar, incubated anaerobically at 30 °C for 72 h, and counted. Enterobacteriaceae were plated on Violet Red Bile Glucose agar, incubated at 37 °C for 24 h, and counted based on characteristic coloration. Coliforms were plated on Violet Red Bile Lactose agar, incubated at 30 °C for 24 h, and counted based on specific coloration. β-glucuronidase-positive *Escherichia coli* were plated on Tryptone Bile X-glucuronide agar, incubated at 44 °C for 18–24 h, and blue-green colonies were counted. Coagulase-positive *Staphylococcus aureus* dilutions were plated on Baird-Parker agar with egg yolk tellurite, incubated at 37 °C for 24–48 h, and confirmed by coagulase test. *Salmonella* spp. were pre-enriched, selectively enriched in Rappaport-Vassiliadis and Selenite Cystine broths, plated on Xylose Lysine Deoxycholate and Hektoen Enteric agars, incubated at 37 °C for 24–48 h, and typical colonies were confirmed with biochemical tests. *Listeria monocytogenes* underwent primary and secondary enrichment, was plated on ALOA and PALCAM agars, incubated at 37 °C for 24–48 h, and typical colonies were counted. Colonies were counted, log_10_-transformed and reported as CFU/g. *Salmonella* spp. and *Listeria monocytogenes* findings were reported as either being present or absent in a 25 g sample.

### 2.6. Lipid Oxidation

Lipid oxidation during storage from day 30 to day 90 was assessed by measuring thiobarbituric acid reactive substances (TBARS), following a modified version of Vyncke’s method [41], as detailed in the recent study of Papatzimos et al. [8]. The sample was homogenised with aqueous trichloroacetic acid containing n-propyl gallate and ethylenediaminetetraacetic acid. Following TBARS extraction, the resulting mixture was filtered, and a portion was combined with aqueous thiobarbituric acid. At the same time, a blank sample was concurrently prepared with trichloroacetic acid and thiobarbituric acid solutions. The absorbance was measured against the blank sample with a UV–VIS spectrophotometer after the mixture was stored overnight in darkness at room temperature. TBARS (TBA number), expressed as mg of malonaldehyde per kg of the sample, were quantified with 1,1,3,3-tetraethoxypropane as a standard.

### 2.7. Active Acidity (pH) and Water Activity (a_w_)

Active acidity (pH) and water activity (a_w_) were measured on storage days 0, 30, 60, and 90, following the procedures outlined in Papatzimos et al. [8]. For pH measurement, a glass electrode equipped with a built-in temperature sensor was used after calibration as per the manufacturer’s instructions. Ten grams of sample were homogenized with 100 mL of distilled water, and the pH was measured by immersing the electrode in the mixture [42].

Water activity (a_w_) was measured on intact sample specimens using a disposable cup filled with the sample and placed in the sample holder. Then the probe, positioned on top of the sample holder, initiated a 4-min measurement cycle. Before measurement was initiated, the covered disposable sample cups were left on the same working area as the probe to adjust to equilibrate to the room temperature.

### 2.8. Colour Evaluation

The colour measurements were performed using the Minolta CR-410 colorimeter (Konica Minolta Company, Osaka, Japan) with a 50-mm measuring area (aperture size) utilizing illuminant source C and 2° standard observer angle on storage days 30, 60, and 90. The light projection tube (CR-A33a, Konica Minolta, Japan) was placed over the aperture port during measurement. Before usage, the colorimeter was calibrated using a white calibration plate (Y = 93.9, x = 0.3136, y = 0.3201). Measurements were conducted on samples that had been removed from the vacuum packaging under and cross-sectioned after an interval of at least 40 min (30 min following package removal and 10 min following cutting) [43]. Sample colour was expressed using the L*a*b* system, where L* denotes luminosity, a* denotes the redness, and b* denotes the yellowness. The following parameters were determined according to the equations presented by Kasapidou et al. [44].

Chroma (colour saturation) = (a*^2^ + b*^2^)^0.5^

Hue angle = [tan^−1^ (b*/a*)] expressed in degrees

All L*, a*, b* values were the instrumental average of three independent measurements collected from random sites across the sample avoiding small areas of severe discoloration.

Total colour difference (Δ*E*_Lab_) between the control and samples in treatments containing hemp seed oil on the same storage day was also determined using the following equation [45].

Δ*E*_Lab_ = [(ΔL*)^2^ + (Δa*)^2^ + (Δb*)^2^]^0.5^

### 2.9. Texture Profile

The texture profile, which mimics the biting action in the mouth, was assessed using the Perten TVT 6700 texturometer paired with TexCal5^®^ software (Perten Instruments, Hägersten, Sweden) on storage days 30, 60 and 90. This evaluation followed a modified version of method 56.01 that is suitable for red meat properties, utilizing a double cycle compression technique. The procedure employed a cylinder probe (P-CY20S, 20 mm diameter) and a 10 kg load cell. The test samples had a height of approximately 25–30 mm. The examined textural characteristics were hardness, springiness, cohesiveness, gumminess, and chewiness. Prior to testing, samples were brought to room temperature.

### 2.10. Sensory Evaluation

The salamis were assessed by panel comprising 10 members working in the food and hospitality industry. The panel members had more than three-year experience in the sensory assessment of meat and meat products as part of their working activities although they had not completed a sensory training course. The development of the questionnaire was based on similar studies and was conducted in collaboration with the panel members to ensure that the language and descriptors were understood and agreed upon by all participants. Sensory evaluation was conducted at the end of the storage period using samples from the second production batch. The edges of the salami were cut off [46], and the remaining salami was sliced into 2 cm thick pieces [18], which were presented monadically on a white china plate. Samples were coded with a 3-digit random number to avoid bias and the presentation of the samples across participants was randomized to prevent artifacts due to the order of sample presentation [47]. The evaluation of the products included overall appearance, colour, odour intensity, odour, taste intensity, taste, tenderness, cohesiveness, oiliness, juiciness, aftertaste and overall impression (acceptability) using a 7-point hedonic scale [18] (1 = dislike very much, 2 = dislike moderately, 3 = dislike slightly, 4 = neither like nor dislike, 5 = like slightly, 6 = like moderately, 7 = like very much).

### 2.11. Statistical Analysis

Data was organized as treatment averages ± standard deviation (SD). The homogeneity of variances was evaluated using the Levene test. One-way analysis of variance was conducted, followed by Tukey’s post-hoc test in instances of homogeneous variances. When homogeneity of variance was not met, the Games-Howell test was used to compare treatments. Statistical significance was considered when the resulting *p*-values were less than 0.05. Data analysis was conducted using SPSS software (version 29.0.0.0, SPSS Inc., Chicago, IL, USA).

## 3. Results and Discussion

### 3.1. Hemp Seed Oil Fatty Acid Composition and Cannabidiol Content

The cannabis oil used was a commercially available food-grade product extracted by cold pressing from industrial hemp (*Cannabis sativa* L.) of the Finola variety. The analysis for fatty acid composition was conducted in an accredited external laboratory and it was provided by the producer. The tetrahydrocannabinol content was determined in an external laboratory. The fatty acid composition of hemp seed oil is presented in Table 3. According to Siano et al. [21] hemp seed oil contains mainly polyunsaturated fatty acids such as linoleic acid (55–57%), alpha-linolenic acid (16%), γ-linolenic acid (2.7%), oleic acid (11%). A similar fatty acid composition was reported in the recent work of Golimowski et al. [48], which examined the fatty acid profile of oil from various hemp seed varieties, including Finola.

The cannabidiol (CBD) content was lower than the values reported by Jang et al. [49] for commercial hemp oil samples sold in Korea, where the CBD content ranged from 6.66 to 63.40 mg/mL. Petrovic’ et al. [50] also reported that the CBD content of commercial-grade hemp seed oil that were either purchased from natural food stores or donated by manufacturers, ranged from 4.18 to 243.68 mg/kg and thus far above the CBD content (1.2 μg/g) of the oil used in the present study. The previous workers related this finding to differences between the hemp varieties. CBD has demonstrated various pharmacological properties, making it one of the most researched phytocannabinoids for potential therapeutic applications in numerous medical conditions [51]. Depending on the hemp variety, it can predominantly produce either THC or CBD. It has been suggested to differentiate cannabis into drug-type (marijuana), which is high in THC, and fiber-type (hemp), which is high in CBD [52].

### 3.2. Proximate Composition and Residual Nitrite Analysis

The moisture, ash, protein, fat, and sodium chloride contents of the samples are presented in Table 4. Except for the sodium chloride content, there are no statistically significant differences in the chemical composition between treatments. In contrast, sodium chloride content significantly differs (*p* < 0.05) between treatments. All treatments resulted in samples with high fat and sodium chloride content. The high fat and sodium chloride content compromise the nutritional value of the fermented salamis. However, fat content influences organoleptic properties such as taste, texture, juiciness, and appearance [53]. Additionally, the sodium chloride content is critical for microbial stability and taste [54].

The sodium nitrite content of the samples exhibited statistically significant differences (*p* < 0.001) between treatments. In the S0, S1 and S2 treatments, the sodium nitrite content decreased to approximately 10% of the initially added amount. Salamis subjected to the S3 treatment displayed very low residual sodium nitrite content, while in samples from the S4 treatment, residual sodium nitrite was not detected. The residual nitrite content in the S3 treatment samples is likely due to the indirect introduction of nitrites from other ingredients such as spices and flavourings [55]. Furthermore, Sindelar [56] reported residual nitrites in commercially available natural or organic meat products in the USA. The difference in residual nitrite content between the S3 and S4 treatment samples, both of which had no added sodium nitrite, is attributed to the difference to the levels of polyphenols and alpha-tocopherol, which significantly reduce nitrite content in meat products [57]. Briefly, nitrite has the ability to be converted into nitrous acid or nitric oxide, which can subsequently react with polyphenols or other endogenous substances, reducing residual nitrites [58]. According to Smeriglio et al. [59], cold-pressed hemp oil from Finola hemp, such as the one used in the present study, has a high content of phenolic compounds, mainly flavonoids and tocopherols.

The sodium nitrite content in meat products is influenced by various factors, which in turn affect the formation of N-nitroso-compounds (NOCs). The formation of NOCs depends on various factors, including the amount of added nitrite, the quality of the meat, the fat content, the processing conditions, the curing treatment, and even how consumers handle the product. Processing factors encompass the use of additives, heating during drying or smoking, storage and curing conditions, and packaging [60].

### 3.3. Fatty Acid Composition and Nutritional Indices

Fatty acid composition of the salamis is presented in Table 5. The fatty acid profile depends mainly on the fatty acid composition of pork fat, the fatty acid composition of hemp seed oil and the fatty acid composition of intramuscular meat fat. Palmitic (C16:0) and stearic (C18:0) acids were the primary saturated fatty acids. Monounsaturated fatty acids consisted mainly of oleic (C18:1 *cis*) and palmitoleic (C16:1) acids, while polyunsaturated fatty acids consisted mainly of linoleic (C18:2 *n-6*) and alpha-linolenic (C18:3 *n-3*) acids. The hemp seed oil fatty acid composition (Table 3) is reflected in the salamis containing hemp seed oil in comparison to samples on the S0 treatment. The levels of linoleic (C18:2 *n-6*) and alpha-linolenic (C18:3 *n-3*) acids significantly increased (*p* < 0.001) as the levels of hemp seed oil substitution increased. Similarly, the levels of oleic acid (C18:1 *cis*) decreased. Samples containing hemp seed oil had significantly lower levels of saturated and monounsaturated fatty acids (*p* < 0.001), while the levels of polyunsaturated fatty acids were significantly higher (*p* < 0.001).

A similar profile was observed in meatballs containing hemp seed oil and in frankfurters containing a gelled emulsion consisting of hemp oil and buckwheat flour, as reported by Montowska et al. [17] and Botella-Martínez et al. [18], respectively. An improvement in the fatty acid composition was also noted in meatloaf products prepared with the addition of hemp seeds [61]. Poultry roasts containing a combination of hemp components such as seeds, flour, and roasted seeds had a favourable fatty acid profile for human health compared to control products [20].

Although the application of vegetable fats, such as olive oil, can influence the fat composition of meat products, the results are not consistent. For example, Del Nobile et al. [62] reported that replacing 60–100% of pork fat with olive oil in Italian-style salami altered the fatty acid composition. However, in other studies on cured sausages, despite replacing 25% of pork fat with olive oil, no changes in the fatty acid composition were observed [63]. In the present study, the addition of hemp seed oil at a relatively low percentage resulted in products with significantly improved fatty acid composition, which has also been corroborated in other studies [17,20].

The ratio of polyunsaturated to saturated fatty acid (PUFA/SFA) was significantly lower (*p* < 0.001) in the samples from the control (S0) treatment (Table 6). The PUFA/SFA ratio is frequently used as an indicator of the nutritional quality of fat. According to the UK guidelines [64] for healthy fat consumption, this ratio should be 0.45 since higher ratios are associated with a decreased risk for cardiovascular disease. The treatments with the closest values to this recommendation are S2 and S4. Additionally, the atherogenic (AI) and the thrombogenic (TI) indices were significantly lower (*p* < 0.001) in the treatments containing hemp seed oil. The values for both indices should be less than 3, and generally lower values are considered better for healthy human nutrition [65]. In detail AI presents the relationship between saturated fatty acids like lauric (C12:0), myristic (C14:0), and palmitic acid (C16:0), which promote atherosclerotic plaque formation, and unsaturated fatty acids, which hinder plaque formation and lower phospholipid and cholesterol levels. The TI refers to the potential of fatty acids to form clots in blood vessels. Finally, the h/H ratio represents the balance between fatty acids that reduce cholesterol and those that raise it, with higher values considered favourable [31]. Botella-Martínez et al. [18] studied also the nutritional indices in frankfurters containing a gelled emulsion consisting of hemp oil and buckwheat flour and reported significantly improved lipid profile indices as the fat substitution level increased.

### 3.4. Microbiological Analysis

The counts of moulds, Enterobacteriaceae, coliforms, *Escherichia coli* β-glucurinidase and *Staphylococcus aureus* did not differ between treatments during the entire study period. In detail, populations were lower than 1.00, 1.00, 1.00, 1.00 and 2.30 log_10_ CFU/g for moulds, Enterobacteriaceae, coliforms, *Escherichia coli* β-glucurinidase and *Staphylococcus aureus* respectively. Additionally, *Salmonella* spp. and *Listeria monocytogenes* were not detected (absence in 25 g) during the entire storage period in any of the analysed samples. The results for the remaining of the examined microorganisms are presented in Table 7. The results show that either partial or entire reduction of sodium nitrite content i.e., treatments S1 to S4, resulted in higher counts of total aerobic microorganisms, yeasts and mesophilic lactic acid bacteria in relation to samples on the S0 treatment. The higher counts of total aerobic microorganisms are related to the starter culture used to improve the quality characteristics of the salamis [46,66]. Lactic acid bacteria were the dominant flora in samples from all treatments and the counts were similar to those of total bacteria microorganisms in accordance with the study of Cenci-Goga et al. [46]. Gonzales-Barron et al. [67] also reported that the populations of total viable bacteria are related to the growth of lactic acid bacteria which rapidly becomes the main microbial group as fermentation procedure was evolving in Portuguese traditional dry fermented sausages. Lactic acid bacteria have a significant role in the fermentation process and overall product safety [68]. Nevertheless, the microbial populations of the tested organisms comply with the hygienic standards set by current regulations [69], for *Listeria monocytogenes* in ready-to-eat food products. Additionally, the product characteristics align with the findings of Magra [70], who studied the microbial profile of commercial samples of fermented salamis produced without a starter culture, and Papatzimos [71], who examined the microbiological parameters of fermented meat products collected from processing plants and retail facilities. In detail, the values for Enterobacteriaceae populations were similar to those reported by Magra [72]. The counts for moulds and yeasts were also within the range (2.97–4.24 log10 CFU/g) found by Magra, while the counts for mesophilic lactic acid bacteria were higher (6.98–7.41 log10 CFU/g) due to the fact that starter cultures were used. Papatzimos [71] reported also absence of *Listeria monocytogenes* in the examined samples of fermented meat products.

In relation to vegetable oils and preservatives in fermented meat products, Bloukas et al. [72] found that partial replacement of pork fat with olive oil in fermented sausages did not impact the levels of lactic acid bacteria, micrococci, and staphylococci. On the other hand, Hospital et al. [73] reported that reducing nitrites/nitrates concentration by 50% would offer the same protection against *Salmonella* as the current maximum permitted levels set by the EU for dry fermented sausages, which aligns with the findings of the current study. Finally, the presence of nitrites in treatments S0, S1, and S2 did not result in a reduction in the counts of Enterobacteriaceae and *Staphylococcus aureus* compared to samples in the S3 and S4 treatments. This indicates that other parameters, such as pH and good manufacturing practices, affect microbiological safety indicators, as reported in the previous study by Gonzales-Barron et al. [67] with fermented sausages.

### 3.5. Lipid Oxidation

**Figure 2:**
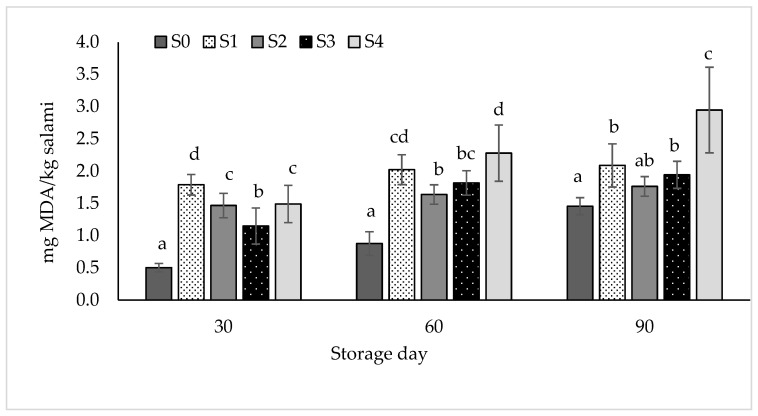
Changes in lipid oxidation of the salamis during storage. S0 = control—NaNO_2_ 100 mg/Kg; S1 = Hemp seed oil 2%—NaNO_2_ 50 mg/Kg; S2 = Hemp seed oil 4%—NaNO_2_ 50 mg/Kg; S3 = Hemp seed oil 2%; S4 = Hemp seed oil 4%. Superscripts a, b, c, d differ at *p* < 0.05 within the same storage period.

Changes in lipid oxidation during preservation of the samples for 90 days under simulated retail conditions are shown in Figure 2. Lipid oxidation increased during storage in all treatments and highly statistically significant differences were observed between treatments (*p* < 0.001) within each storage period. However, it was observed that there was no consistent pattern for the extent of lipid oxidation in the samples containing sodium nitrite and hemp seed oil or hemp seed oil alone. As shown significant differences (*p* < 0.05) were found between salamis in treatments S1 and S3 on storage day 30 whereas there were no differences (*p* > 0.05) between samples on the same treatments on storage days 60 and 90. On the other hand, there were no significant differences between samples on the S2 and S4 treatments on storage day 30 while significant differences (*p* < 0.05) were observed between the same treatments on storage days 60 and 90. Montowska et al. [17] also found a non-constant pattern in the changes in lipid oxidation during a 17-day storage period in meatballs containing hemp seed oil at similar levels to the present study, i.e., 2.5% and 4.2%. On the other hand, Botella-Martínez et al. [18] reported no statistically significant differences in the TBA number in frankfurters where pork fat was partially replaced (25–100%) with a gelled emulsion consisting of hemp oil and buckwheat flour. They linked this positive outcome to the encapsulated oil droplets within the gel matrix, which functioned as a protective shield against oxidation [74]. Severini et al. [75] reported similar fluctuations in the degree of oxidation in salami products with partial substitution of pork fat with olive oil and attributed this to a combination of aldehydes with other components as well as the loss of volatile aldehydes.

Coutinho de Oliveira et al. [76] observed lower lipid oxidation in mortadella-type sausages containing reduced amounts of the essential oil of *Satureja montana* L. and sodium nitrite compared to the same products containing higher amounts of *Satureja montana* L. and sodium nitrite. These researchers related this effect to a better synergistic action between the antioxidant substances in the essential oil and sodium nitrite.

The higher oxidation observed in the samples of the treatments containing hemp seed oil was attributed to the presence of easily oxidizable unsaturated fatty acids. Bloukas et al. [72] and Severini et al. [75] also reported increased lipid oxidation in meat products containing olive oil as a substitute for pork fat, attributing this increase to unsaturated fatty acids present in olive oil. Regarding the oxidation observed during the preservation of the samples, it is noted that the combination of a high residual oxygen concentration and the high oxygen permeability of the packaging material can lead to increased oxidation during storage [77].

TBA number, which describes the extent of lipid oxidation, was higher than 1 mg malonaldehyde/kg sample, a value considered the threshold for perceiving the taste of rancidity for meat products [42]. Greene and Cumuze [78] determined that a TBA value in the range of 0.6 to 2.0 is the threshold at which an inexperienced panel can detect oxidized flavours in ground beef. Finally, Domínguez et al. [79] indicated that the acceptable threshold for perceiving rancidity in meat and meat products is between 2 and 2.5 mg malonaldehyde/kg sample of sample. The odour perception of rancidity in meat products depends on other factors such as the presence of flavouring agents. However, hemp seed oil has a strong flavour which could mask the taste of rancidity. In any case, the consumption of oxidized lipids should be avoided as it has been linked to adverse health effects due to the onset of oxidative stress, which contributes to the development of chronic diseases. The oxidation of lipids produces potentially harmful substances that are linked to inflammatory diseases, cancer, atherosclerosis, and the aging process. These harmful substances can be introduced into the body through diet and can also form within the body during the digestion of lipids [80]. Furthermore, lipid oxidation in meat products affects also characteristics such as colour and texture affecting product acceptability [79].

### 3.6. Active Acidity (pH) and Water Activity (a_w_)

Active acidity (pH) and water activity (a_w_) of the salami during storage are shown in Table 8. The pH values of the samples containing only hemp seed oil i.e., treatments S3 and S4 were lower than those of the control and the treatments containing both hemp seed oil and sodium nitrite i.e., treatments S0, S1 and S2. Statistically significant differences (*p* < 0.01) in the pH were observed during the entire storage period between the different treatments. Montowska et al. [17] reported that neither the amount nor the storage duration affected the pH values of meatballs containing hemp seed oil. Botella-Martínez et al. [18] reported statistically significant differences in the pH values of frankfurters where pork fat was partially replaced (25% and 50%) with a gelled emulsion consisting of hemp oil and buckwheat flour. Finally, in a similar study, where olive oil was used statistically significant differences were also observed between samples containing different levels of olive oil during storage of the salami samples [75].

Water activity (a_w_) decreased during storage and statistically significant differences (*p* < 0.001) were observed between treatments. In general, during the entire storage period, salamis from treatments S2 and S3, which contained hemp seed oil and nitrites, had lower water activity values compared to samples from the control (S0) treatment. These findings show that the addition of hemp seed oil can significantly reduce water activity, even when sodium nitrite levels are substantially decreased. Samples containing the higher level of hemp seed oil alone i.e., the S4 treatment had similar levels of water activity with the samples on the control (S0) treatment. Montowska et al. [17] reported higher water activity values in meatballs containing increased levels of hemp oil i.e., 7.5% in relation to samples that did not contain hemp oil. In the latter study, lower levels of hemp oil did not affect the water activity. Similarly, in the previous reported study of Botella-Martínez et al. [18] higher water activity values were observed as the fat substitution level increased. Severini et al. [75] higher water activity in salami samples containing olive oil and attributed the increased values to the lower degree of drying during the ripening of the samples.

The pH values of the salamis fell within the range of 4.3 to 6.3 reported by Herranz et al. [81] for salamis produced across various European countries. However, the water activity values were outside the range of 0.790 to 0.960 reported by the same researchers. These differences were attributed to variations in product formulation, including the type and amount of meat, fat, sugar, starter culture, and the ripening process.

Regarding the growth of microorganisms during the preservation of the samples, the samples can be classified as shelf stable i.e., not requiring refrigeration in terms of active acidity and water activity, as they have pH values < 5.2 and water activity < 0.91 [82]. According to the previous researchers, the shelf life of such products is not constrained by bacterial growth but by chemical or physical deterioration, particularly rancidity and discoloration.

### 3.7. Colour Evaluation

Changes in colour characteristics from storage day 30 to storage day 90 are shown in Table 9. The results show treatment and storage period significantly affect the colour characteristics of the samples. Briefly, lightness (L*) increased over time for most treatments, with significant differences (*p* < 0.001) between treatments at all storage times. Redness (a*) decreased over time, with significant differences (*p* < 0.001) between treatments at day 30 and 60 day, but these differences became non-significant (*p* > 0.05) by the 90th day of storage. Highly significant differences (*p* < 0.01) were observed in yellowness (b*) values for storage days 60 and 90. Chroma decreased over time, with significant differences (*p* < 0.001–*p* < 0.05) among treatments at all storage periods. Finally, hue angle showed the most variability, with significant differences (*p* < 0.001) at 60 and 90 days but not at 30 days. According to the recent guidelines for colour measurements by King et al. [45] hue angle is a useful indicator for shifts in colour over time toward discoloration. Additionally, larger values are associated with less red and more metmyoglobin.

Treatments containing sodium nitrite i.e., S0, S1 and S3 had a more intense red colour (a*) indicating the important role of nitrite salts in the formation of the red colour in fermented products [83]. However, the redness and chroma values indicate that the addition of hemp seed oil can result in a significant reduction in sodium nitrite, as the salamis from treatments S1 and S2 had a similar colour to that of the control (S0) treatment, which contained 100 mg/kg sodium nitrite. Hemp seed oil affected also significantly the redness and yellowness values of meatballs and frankfurters containing either hemp seed oil or a gelled emulsion consisting of hemp oil and buckwheat flour respectively [17,19].

In similar studies in cured meat products such as sausages and salamis, where pork fat was partially substituted with olive oil, lower redness values were also found [72,76]. Additionally, differences in the colour parameters due to substitution of animal fat with plant oil emulsions has also been reported by other researchers [84] and it was related to the colours of the oils and the difference in globule diameter of the oil emulsions which allows greater light reflection.

**Figure 3:**
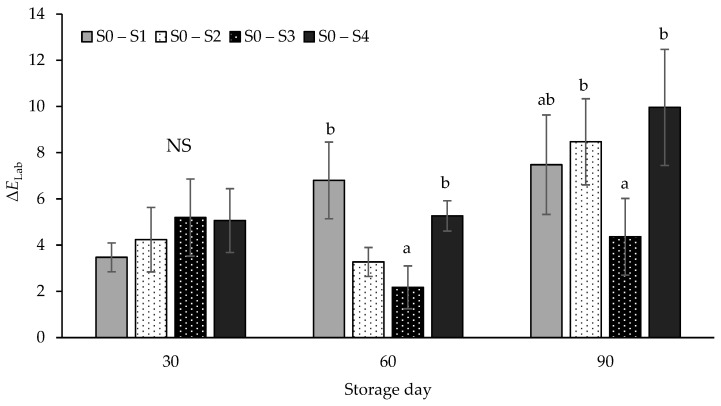
Total colour difference (ΔE_Lab_) between control (S0) and samples containing hemp seed oil (treatments S1 to S4). S0 = control—NaNO_2_ 100 mg/Kg; S1 = Hemp seed oil 2%—NaNO_2_ 50 mg/Kg; S2 = Hemp seed oil 4%—NaNO_2_ 50 mg/Kg; S3 = Hemp seed oil 2%; S4 = Hemp seed oil 4%. NS = Non-significant. Superscripts a, b differ at *p* < 0.05 within the same storage period.

The total colour difference (ΔELab) between samples from the S0 treatment and those from treatments S2 to S4 containing hemp seed oil is shown in Figure 3. According to Tomasevic et al. [85], the total colour difference (ΔELab) is used as an index to assess the variation between samples from groups different groups. Higher ΔELab values indicate greater colour differences between treated and control samples.

There were no significant differences (*p* > 0.05) in ΔELab values between the control samples and those containing hemp seed oil on storage day 30. However, significant differences were observed on days 60 (*p* < 0.001) and 90 (*p* < 0.01). On these days, the lowest colour difference values were observed between the control samples and those from the S3 treatment, indicating that higher levels of hemp seed oil contribute favorably to colour stability.

According to Brainard [86], total colour difference values ranging from 2 to 10 are instantly perceptible, similar to the values observed in the present study throughout the entire examination period. It should be noted that the ΔELab values resulting in perceptible differences are influenced by the characteristics of the products, and there is no specific information on threshold values for meat products [87]. Botella-Martínez et al. [18] reported also statistically significant differences in the ΔELab values of frankfurters where pork fat was partially replaced with a gelled emulsion made of hemp oil and buckwheat flour. They attributed this difference to the greenish-yellow colour of the emulsion.

### 3.8. Texture Profile

Texture profile analysis (TPA) of the salamis during storage is presented in Table 10. Texture is a very significant characteristic of salami products since it is related to its sliceability. TPA in meat products such as salamis involves measuring several key parameters to evaluate their textural quality. These parameters include hardness, springiness, cohesiveness, gumminess, and chewiness [61,88]. In detail, hardness is defined as the peak force needed to initially compress the product. Springiness (or elasticity) refers to the ability of the product to recover its original shape after being compressed. Cohesiveness measures how well the product withstands a second deformation compared to the first. Gumminess is calculated by multiplying product hardness and cohesiveness. Finally, chewiness is derived from the product gumminess and springiness, representing the energy required to chew the product [89,90].

The addition of hemp seed oil and/or sodium nitrite significantly affected (*p* < 0.001) the texture profile parameters during the entire study period. The results indicate that the inclusion of hemp seed oil and varying levels of sodium nitrite significantly affect the textural properties of salamis over the storage period. Treatments with higher sodium nitrite content (S0) consistently showed greater hardness, gumminess, and chewiness. Conversely, samples with higher hemp seed oil content (S4) demonstrated lower values in these parameters, suggesting that hemp seed oil can be used to modulate the texture of salamis, potentially producing a softer product that remains acceptable to consumers. The data also highlight the importance of balancing nitrite and hemp seed oil levels to maintain desired textural qualities over time.

The texture profile of the meat products depends on the product type and its composition. In this respect, the effect of adding hemp seed oil should be examined in relation to similar products, such as sausages and salamis. Botella-Martínez et al. [18] reported differences in the textural properties of frankfurters containing a gelled emulsion made of hemp oil and buckwheat flour but they related these to the specific characteristics of the emulsion and its effect in the meat matrix.

### 3.9. Sensory Evaluation

**Figure 4:**
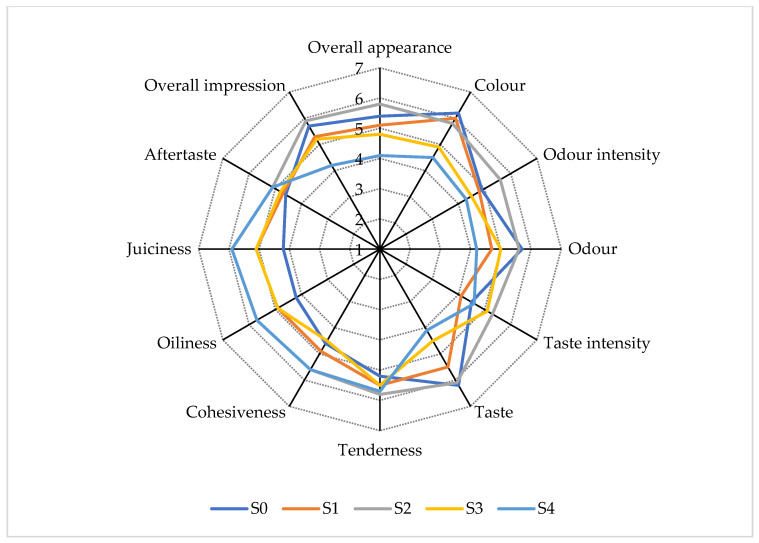
Taste panel scores for the salamis. S0 = control—NaNO_2_ 100 mg/Kg; S1 = Hemp seed oil 2%—NaNO_2_ 50 mg/Kg; S2 = Hemp seed oil 4%—NaNO_2_ 50 mg/Kg; S3 = Hemp seed oil 2%; S4 = Hemp seed oil 4%.

The sensory evaluation results of the salamis at the end of the storage period are shown in Figure 4. Highly significant differences (*p* < 0.001) between treatments were observed for colour, flavour, oiliness, juiciness, and overall acceptability. Significant differences (*p* < 0.01) were found in overall appearance, odour, and cohesiveness. Differences (*p* < 0.05) were noted in odour intensity, while no significant differences (*p* > 0.05) were observed for flavour intensity, tenderness, and aftertaste.

A detailed examination of the multiple comparisons between treatments revealed distinct categories based on sensory evaluation scores. First, samples from the S4 treatment were significantly different (*p* < 0.05) from those of the other treatments. Another category included samples from treatments containing sodium nitrite (S0, S1, and S2). Finally, samples from the S3 treatment fell between the first two categories.

Regarding overall appearance and acceptability, the highest scores were found for samples in the S2 treatment. The color and taste values in this treatment were similar to those recorded for treatments S0 and S1. The addition of hemp seed oil positively affected juiciness, and no undesirable effect on oiliness was observed. The lower taste scores in samples containing hemp seed alone are related to the increased lipid oxidation levels, which lead to the development of unpleasant odours and flavours [78]. Hemp seed has a pleasant nutty taste, and roasting has been reported to enhance the sweetness of hemp seed oil [91,92], explaining the higher scores for samples in the S2 treatment.

Montowska et al. [17] reported higher scores for colour intensity, juiciness, and taste in meatballs containing hemp oil. Additionally, juiciness and hardness were not affected in frankfurters containing a gelled emulsion made of hemp oil and buckwheat flour [18]. Finally, similar scores for both taste and odour intensity were found in poultry roasts enriched with and without hemp components [20]. Despite the significant differences in most of the traits examined, samples from all groups received scores above the acceptability limit (score = 4) for all parameters, indicating that the addition of hemp seed oil in the salamis was positively received by the panelists.

## 4. Conclusions

This study investigated the effect of hemp seed oil addition in combination with sodium nitrite on the nutritional composition and shelf life of fermented salamis. The addition of hemp oil did not affect proximate composition, while it significantly improved the nutritional indices for fatty acid composition. Microbial growth was not affected in all treatments. Active acidity (pH) and water activity (a_w_) were significantly affected by the addition of hemp seed oil and/or sodium nitrite, but without a negative impact on the shelf life of the salamis. However, the colour of the products, in relation to redness (a*) and colour saturation (Chroma), had lower values throughout the storage period in the treatments containing only hemp seed oil. The addition of hemp seed oil led to higher levels of lipid peroxidation, which was reduced when combined with sodium nitrite. Hemp seed oil and varying levels of sodium nitrite significantly affect salami texture during storage. Higher sodium nitrite content increases hardness, gumminess, and chewiness, while higher hemp seed oil content softens the product. Sensory evaluation revealed consumer acceptance of salamis containing hemp seed oil. Considering the variations in colour and lipid oxidation over the storage period, it is recommended to limit the storage of the samples to 60 days, as partial or entire replacement of nitrites with hemp seed oil does not fulfill all the functions of nitrites in meat products.

In summary, hemp seed oil has the potential to be used as a value-added food ingredient to improve the nutritional value of fermented meat products and produce healthier products in combination with reduced sodium nitrite content.

## Figures and Tables

**Table 1:** Ingredient composition of the salamis in relation to treatment.

Ingredient	Treatment				
	S0	S1	S2	S3	S4
Pork meat (max fat content 5.0%) (g/100 g)	50	50	50	50	50
Beef meat (max fat content 4.0%) (g/100 g)	22	22	22	22	22
Pork back fat (g/100 g)	22	20	18	20	18
Hemp seed oil (g/100 g)	0	2	4	2	4
Sodium nitrite (NaNO_2_) (mg/Kg)	100	50	50	0	0
Dextrose (g/100 g)	0.085	0.085	0.085	0.085	0.085
Sodium chloride (NaCl) (g/100 g)	1.10	1.10	1.10	1.10	1.10
Starter culture (*Lactobacillus sakei*, *Staphylococcus carnosus* spp.) (g/100 g)	0.004	0.004	0.004	0.004	0.004
Spice mix (black pepper, mustard, onion, nutmeg, coriander) (g/100 g)	0.025	0.025	0.025	0.025	0.025

S0 = control—NaNO_2_ 100 mg/Kg; S1 = Hemp seed oil 2%—NaNO_2_ 50 mg/Kg; S2 = Hemp seed oil 4%—NaNO_2_ 50 mg/Kg; S3 = Hemp seed oil 2%; S4 = Hemp seed oil 4%.

**Table 2:** Processing conditions in the ripening chamber.

Day	Temperature (°C)	Relative Humidity (%)	Air Movement (m/s)
Fermentation			
0	22	95	0.5–0.7
1	20.5	93	0.5–0.7
2	19	90	0.5–0.7
3	18	88	0.5–0.7
4	17	85	0.5–0.7
5	16	82	0.5–0.7
6	15	80	0.5–0.7
Ripening			
7–18	15	80	0.05–0.1

**Table 3:** Fatty acid composition (g/100 g) and cannabidiol (μg/g) content of hemp seed oil.

Variable	Content
Fatty acid	
C16:0	6.61
C18:0	2.89
C20:0	0.11
C22:0	1.05
C24:0	0.19
C16:1	0.11
C18:1 *cis*	13.2
C20:1	0.14
C18:2 *n-6*	53.9
C18:3 *n-3*	19.5
C20:3 *n-3*	<0.1
C20:3 *n-6*	<0.1
C20:4 *n-6*	<0.1
C20:5 *n-3*	<0.1
C22:6 *n-3*	<0.1
Cannabidiol	1.2

**Table 4:** Proximate composition of salamis (mean values ± SD).

Variable	Treatment					Significance
	S0	S1	S2	S3	S4	
Moisture (g/100 g)	28.58 ± 10.539	29.52 ± 10.4	28.82 ± 7.568	28.32 ± 7.27	27.07 ± 8.223	NS
Ash (g/100 g)	5.03 ± 0.265	5.37 ± 0.566	5.12 ± 0.659	4.9 ± 0.254	5.18 ± 0.507	NS
Protein (g/100 g)	25.54 ± 3.216	27.25 ± 1.262	26.36 ± 2.468	25.54 ± 1.872	23.93 ± 1.448	NS
Fat (g/100 g)	40.10 ± 6.761	37.67 ± 9.661	39.33 ± 5.25	41 ± 60.839	42.38 ± 5.281	NS
Sodium Chloride (g/100 g)	3.32 ± 0.277	3.89 ^b^ ± 0.284	3.30 ± 0.285	3.04 ^a^ ± 0.214	3.11 ^a^ ± 0.487	*
Sodium Nitrite (mg/kg)	11.71 ^c^ ± 0.391	5.06 ^b^ ± 1.161	5.59 ^b^ ± 1.310	1.13 ^a^ ± 0.841	0.00 ^a^ ± 0.000	***

S0 = control—NaNO_2_ 100 mg/Kg; S1 = Hemp seed oil 2%—NaNO_2_ 50 mg/Kg; S2 = Hemp seed oil 4%—NaNO_2_ 50 mg/Kg; S3 = Hemp seed oil 2%; S4 = Hemp seed oil 4%. NS = Non-significant; * = *p* < 0.05; *** = *p* < 0.001; Superscripts a, b, c differ at *p* < 0.05.

**Table 5:** Salami fatty acid composition (% of total identified fatty acids) (mean values ± SD).

Variable	Treatment					Significance
	S0	S1	S2	S3	S4	
Fatty acid						
C10:0	0.10 ± 0.006	0.09 ± 0.004	0.09 ± 0.004	0.09 ± 0.003	0.09 ± 0.005	NS
C12:0	0.09 ± 0.021	0.10 ± 0.013	0.09 ± 0.011	0.10 ± 0.005	0.10 ± 0.034	NS
C14:0	1.76 ^b^ ± 0.050	1.69 ^ab^ ± 0.080	1.59 ^a^ ± 0.047	1.64 ^ab^ ± 0.023	1.61 ^a^ ± 0.100	*
C14:1	0.16 ± 0.007	0.17 ± 0.034	0.14 ± 0.016	0.14 ± 0.016	0.16 ± 0.056	NS
C15:0	0.10 ± 0.005	0.10 ± 0.014	0.09 ± 0.005	0.09 ± 0.005	0.10 ± 0.015	NS
C15:1	0.07 ± 0.009	0.06 ± 0.005	0.07 ± 0.015	0.07 ± 0.019	0.08 ± 0.035	NS
C16:0	25.69 ^c^ ± 0.411	24.8 ^bc^ ± 0.378	24.2 ^ab^ ± 0.408	24.73 ^b^ ± 0.347	23.56 ^a^ ± 0.562	***
C16:1	2.76 ^b^ ± 0.095	2.60 ^ab^ ± 0.047	2.51 ^a^ ± 0.019	2.63 ^ab^ ± 0.033	2.60 ^ab^ ± 0.197	*
C17:0	0.44 ± 0.037	0.41 ± 0.089	0.41 ± 0.096	0.36 ± 0.060	0.37 ± 0.078	NS
C17:1	0.38 ± 0.083	0.43 ± 0.075	0.36 ± 0.046	0.42 ± 0.012	0.44 ± 0.018	NS
C18:0	11.7 ± 0.799	11.47 ± 0.542	10.89 ± 0.37	11.05 ± 0.518	10.43 ± 0.84	NS
C18:1 *trans*	0.17 ± 0.146	0.10 ± 0.027	0.18 ± 0.123	0.37 ± 0.222	0.24 ± 0.302	NS
C18:1 *cis*	43.13 ^c^ ± 0.588	42.24 ^bc^ ± 0.271	41.12 ^a^ ± 0.367	41.49 ^ab^ ± 0.069	40.6 ^a^ ± 0.706	***
C18:1, *trans 11* (VA)	0.16 ± 0.025	0.15 ± 0.028	0.14 ± 0.034	0.16 ± 0.031	0.14 ± 0.036	NS
C18:2 *trans*	0.07 ± 0.027	0.06 ± 0.017	0.05 ± 0.008	0.06 ± 0.005	0.06 ± 0.007	NS
C18:2 *n-6*	10.34 ^a^ ± 1.181	12.00 ^ab^ ± 0.644	13.97 ^cd^ ± 0.812	12.94 ^bc^ ± 0.397	14.95 ^d^ ± 0.794	***
C18:3 *n-6*	0.17 ^a^ ± 0.094	0.33 ^b^ ± 0.064	0.39 ^bc^ ± 0.055	0.30 ^ab^ ± 0.047	0.49 ^c^ ± 0.073	***
C18:3 *n-3*	1.02 ^a^ ± 0.326	1.53 ^ab^ ± 0.191	2.03 ^bc^ ± 0.290	1.68 ^b^ ± 0.163	2.28 ^c^ ± 0.162	***
C18:2 *cis-9 trans-11* (CLA)	0.26 ± 0.053	0.27 ± 0.036	0.30 ± 0.036	0.24 ± 0.065	0.31 ± 0.060	NS
C20:2	0.64 ± 0.053	0.66 ± 0.027	0.59 ± 0.023	0.65 ± 0.053	0.60 ± 0.038	NS
C21:0	0.37 ± 0.023	0.35 ± 0.036	0.34 ± 0.013	0.37 ± 0.029	0.33 ± 0.016	NS
C20:3 *n-6*	0.08 ± 0.005	0.07 ± 0.007	0.07 ± 0.007	0.07 ± 0.005	0.07 ± 0.008	NS
C20:4	0.25 ± 0.029	0.21 ± 0.037	0.22 ± 0.044	0.23 ± 0.03	0.22 ± 0.037	NS
C20:3 *n-3*	0.09 ± 0.005	0.09 ± 0.007	0.09 ± 0.006	0.1 ± 0.005	0.09 ± 0.004	NS
C24:0	0.08 ± 0.006	0.07 ± 0.009	0.07 ± 0.011	0.07 ± 0.009	0.07 ± 0.009	NS
C24:1	0.08 ± 0.005	0.07 ± 0.011	0.07 ± 0.012	0.08 ± 0.010	0.08 ± 0.012	NS
Lipid class						
SFA ^1^	40.28 ^c^ ± 1.039	39.04 ^bc^ ± 0.58	37.72 ^ab^ ± 0.783	38.47 ± 0.773	36.62 ^a^ ± 1.167	***
MUFA ^2^	46.86 ^c^ ± 0.662	45.79 ^bc^ ± 0.384	44.56 ^ab^ ± 0.470	45.31 ^ab^ ± 0.192	44.29 ^a^ ± 1.052	***
PUFA ^3^	12.62 ^a^ ± 1.564	14.91 ^ab^ ± 0.900	17.44 ^cd^ ± 1.145	16.00 ^bc^ ± 0.640	18.80 ^d^ ± 0.920	***
*n-3*	1.11 ^a^ ± 0.327	1.61 ^ab^ ± 0.198	2.14 ^cd^ ± 0.283	1.77 ^bc^ ± 0.165	2.38 ^d^ ± 0.1410	***
*n-6*	11.52 ^a^ ± 1.238	13.31 ^ab^ ± 0.706	15.31 ^cd^ ± 0.863	14.23 ^bc^ ± 0.496	16.42 ^d^ ± 0.859	***

S0 = control—NaNO_2_ 100 mg/Kg; S1 = Hemp seed oil 2%—NaNO_2_ 50 mg/Kg; S2 = Hemp seed oil 4%—NaNO_2_ 50 mg/Kg; S3 = Hemp seed oil 2%; S4 = Hemp seed oil 4%. 1 = Saturated fatty acids; 2 = monounsaturated fatty acids; 3 = polyunsaturated fatty acids; NS = Non-significant; * = *p* < 0.05; *** = *p* < 0.001; Superscripts a, b, c, d differ at *p* < 0.05.

**Table 6:** Salami nutritional indices in relation to healthy fat consumption (mean values ± SD).

Index	Treatment					Significance
	S0	S1	S2	S3	S4	
PUFA/SFA ^1^	0.32 ^a^ ± 0.048	0.39 ^ab^ ± 0.029	0.47 ^c^ ± 0.041	0.42 ^bc^ ± 0.025	0.52 ^c^ ± 0.037	***
AI ^2^	0.56 ^c^ ± 0.017	0.53 ^bc^ ± 0.014	0.50 ^ab^ ± 0.016	0.52 ^b^ ± 0.012	0.48 ^a^ ± 0.020	***
TI ^3^	1.33 ^c^ ± 0.053	1.27 ^bc^ ± 0.031	1.21 ^ab^ ± 0.036	1.25 ± 0.039	1.16 ^a^ ± 0.058	***
h/H ^4^	2.03 ^a^ ± 0.067	2.16 ^ab^ ± 0.051	2.27 ^bc^ ± 0.068	2.18 ^ab^ ± 0.051	2.36 ^c^ ± 0.104	***

S0 = control—NaNO_2_ 100 mg/Kg; S1 = Hemp seed oil 2%—NaNO_2_ 50 mg/Kg; S2 = Hemp seed oil 4%—NaNO_2_ 50 mg/Kg; S3 = Hemp seed oil 2%; S4 = Hemp seed oil 4%. 1 = Polyunsaturated fatty acids/Saturated fatty acids ratio; 2 = atherogenicity index; 3 = thrombogenicity index; 4 = hypocholesterolaemic: hypercholesterolaemic ratio; *** = *p* < 0.001; Superscripts a, b, c differ at *p* < 0.05.

**Table 7:** Changes in the microbiological profile (log_10_ CFU/g) during refrigerated storage of the salamis.

Storage Day	Treatment				
	S0	S1	S2	S3	S4
Total bacterial microorganisms					
30	7.76	9.93	9.38	8.74	9.40
60	8.04	10.20	9.62	9.00	9.64
90	8.36	10.48	9.93	9.32	9.95
Yeasts					
30	2.81	4.23	2.65	2.90	4.15
60	3.08	4.48	2.91	3.18	4.41
90	3.40	4.76	3.28	3.46	4.76
Mesophilic lactic acid bacteria					
30	7.62	8.90	7.99	7.40	9.30
60	7.98	10.08	9.45	8.30	8.34
90	8.18	10.34	8.90	9.26	9.28

S0 = control—NaNO_2_ 100 mg/Kg; S1 = Hemp seed oil 2%—NaNO_2_ 50 mg/Kg; S2 = Hemp seed oil 4%—NaNO_2_ 50 mg/Kg; S3 = Hemp seed oil 2%; S4 = Hemp seed oil 4%.

**Table 8:** Changes in active acidity (pH) and water activity (a_w_) during storage of the salamis (mean values ± SD).

Storage Day	Treatment					Significance
	S0	S1	S2	S3	S4	
Active acidity (pH)						
0	4.95 ^c^ ± 0.013	4.89 ^bc^ ± 0.018	4.86 ^bc^ ± 0.145	4.71 ^ab^ ± 0.018	4.79 ^a^ ± 0.006	**
30	4.93 ^c^ ± 0.010	4.87 ^bc^ ± 0.013	4.84 ^bc^ ± 0.148	4.69 ^ab^ ± 0.015	4.78 ^a^ ± 0.006	**
60	4.88 ^c^ ± 0.035	4.84 ^bc^ ± 0.047	4.82 ^bc^ ± 0.136	4.67 ^ab^ ± 0.018	4.73 ^a^ ± 0.027	**
90	4.88 ^c^ ± 0.018	4.83 ^bc^ ± 0.050	4.81 ^bc^ ± 0.139	4.66 ^ab^ ± 0.031	4.71 ^a^ ± 0.018	**
Water activity (a_w_)						
0	0.789 ^c^ ± 0.0223	0.752 ^b^ ± 0.0078	0.703 ^a^ ± 0.005	0.783 ^c^ ± 0.019	0.741 ^b^ ± 0.0077	***
30	0.787 ^c^ ± 0.0219	0.749 ^b^ ± 0.009	0.701 ^a^ ± 0.005	0.779 ^c^ ± 0.0152	0.740 ^b^ ± 0.0074	***
60	0.785 ^c^ ± 0.0232	0.742 ^b^ ± 0.0111	0.699 ^a^ ± 0.0051	0.776 ^c^ ± 0.0137	0.737 ^b^ ± 0.0088	***
90	0.779 ^d^ ± 0.0218	0.741 ^bc^ ± 0.0126	0.695 ^a^ ± 0.0052	0.764 ^cd^ ± 0.0148	0.723 ^ab^ ± 0.0196	***

S0 = control—NaNO_2_ 100 mg/Kg; S1 = Hemp seed oil 2%—NaNO_2_ 50 mg/Kg; S2 = Hemp seed oil 4%—NaNO_2_ 50 mg/Kg; S3 = Hemp seed oil 2%; S4 = Hemp seed oil 4%. ** = *p* < 0.01; *** = *p* < 0.001; Superscripts a, b, c, d differ at *p* < 0.05.

**Table 9:** Instrumental colour changes during refrigerated storage of the salamis (mean values ± SD).

Storage Day	Treatment					Significance
	S0	S1	S2	S3	S4	
Lightness (L*)						
30	34.48 ^b^ ± 2.807	35.05 ^b^ ± 0.944	30.46 ^a^ ± 1.383	36.28 ^a^ ± 1.812	35.36 ^b^ ± 1.99	***
60	36.71 ^cd^ ± 0.709	30.23 ^a^ ± 1.545	34.43 ^b^ ± 0.437	35.77 ^bc^ ± 1.384	38.39 ^d^ ± 0.944	***
90	30.91 ^b^ ± 2.043	37.45 ^c^ ± 1.01	37.84 ^cd^ ± 1.21	28.01 ^a^ ± 0.743	40.22 ^d^ ± 1.65	***
Redness (a*)						
30	10.58 ^b^ ± 0.563	9.73 ^b^ ± 1.179	10.11 ^b^ ± 0.05	7.99 ^a^ ± 0.324	7.82 ^a^ ± 0.202	***
60	8.64 ^b^ ± 0.814	8.33 ^b^ ± 0.752	8.62 ^b^ ± 0.909	7.98 ^b^ ± 0.173	6.13 ^a^ ± 1.633	***
90	7.43 ± 2.543	7.34 ± 0.98	7.36 ± 0.869	6.44 ± 0.245	6.05 ± 0.143	NS
Yellowness (b*)						
30	0.50 ± 0.640	0.56 ± 0.513	1.12 ± 0.094	1.22 ± 2.162	0.37 ± 1.02	NS
60	−0.16 ^ab^ ± 1.37	−0.85 ^a^ ± 1.016	1.47 ^b^ ± 0.636	0.89 ^ab^ ± 1.383	3.36 ^c^ ± 0.517	***
90	−0.49 ^a^ ± 1.006	0.65 ^a^ ± 2.743	3.79 ^b^ ± 1.337	−1.51 ^a^ ± 0.426	0.55 ^a^ ± 0.943	***
Chroma (Colour saturation)						
30	10.61 ^b^ ± 0.533	9.76 ^b^ ± 1.147	10.18 ^b^ ± 0.054	8.32 ^a^ ± 0.377	7.89 ^a^ ± 0.239	***
60	8.74 ^b^ ± 0.787	8.43 ^ab^ ± 0.749	8.77 ^b^ ± 0.793	8.13 ^ab^ ± 0.289	7.02 ^a^ ± 1.557	*
90	7.54 ^ab^ ± 2.419	7.77 ^ab^ ± 1.141	8.35 ^b^ ± 1.027	6.63 ^ab^ ± 0.200	6.14 ^a^ ± 0.187	*
Hue angle						
30	2.83 ± 3.597	3.59 ± 3.432	6.31 ± 0.516	8.36 ± 15.01	2.64 ± 7.33	NS
60	−1.54 ^ab^ ± 9.157	−6.00 ^a^ ± 6.767	10.03 ^b^ ± 5.094	6.13 ^ab^ ± 9.654	29.42 ^c^ ± 5.932	***
90	−6.47 ^a^ ± 11.164	2.57 ^a^ ± 20.32	26.83 ^b^ ± 8.532	−13.25 ^a^ ± 3.865	4.99 ^a^ ± 8.808	***

S0 = control—NaNO_2_ 100 mg/Kg; S1 = Hemp seed oil 2%—NaNO_2_ 50 mg/Kg; S2 = Hemp seed oil 4%—NaNO_2_ 50 mg/Kg; S3 = Hemp seed oil 2%; S4 = Hemp seed oil 4%. NS = Non-significant; * = *p* < 0.05; *** = *p* < 0.001; Superscripts a, b, c, d differ at *p* < 0.05.

**Table 10:** Changes in texture profile during refrigerated storage of the salamis (mean values ± SD).

Storage Day	Treatment					Significance
	S0	S1	S2	S3	S4	
Hardness (Ν)						
30	66.59 ^e^ ± 1.164	46.67 ^c^ ± 0.554	34.83 ^a^ ± 0.011	63.13 ^d^ ± 0.660	45.25 ^b^ ± 0.462	***
60	36.81 ^e^ ± 0.365	34.27 ^d^ ± 0.657	23.98 ^a^ ± 0.479	25.26 ^b^ ± 0.503	28.32 ^c^ ± 0.628	***
90	53.65 ^e^ ± 0.012	29.03 ^c^ ± 1.580	24.47 ^b^ ± 0.481	34.14 ^d^ ± 0.339	18.37 ^a^ ± 0.265	***
Springiness						
30	0.48 ^c^ ± 0.003	0.53 ^d^ ± 0.005	0.35 ^a^ ± 0.001	0.43 ^b^ ± 0.005	0.56 ^e^ ± 0.008	***
60	0.37 ^c^ ± 0.006	0.31 ^a^ ± 0.009	0.52 ^e^ ± 0.006	0.32 ^b^ ± 0.007	0.46 ^d^ ± 0.005	***
90	0.42 ^b^ ± 0.005	0.43 ^c^ ± 0.001	0.42 ^b^ ± 0.005	0.42 ^b^ ± 0.005	0.32 ^a^ ± 0.005	***
Cohesiveness						
30	0.41 ^c^ ± 0.008	0.38 ^a^ ± 0.005	0.40 ^b^ ± 0.001	0.43 ^d^ ± 0.005	0.41 ^bc^ ± 0.005	***
60	0.45 ^d^ ± 0.005	0.33 ^b^ ± 0.007	0.53 ^e^ ± 0.011	0.28 ^a^ ± 0.006	0.37 ^c^ ± 0.009	***
90	0.46 ^d^ ± 0.001	0.37 ^b^ ± 0.021	0.36 ^b^ ± 0.008	0.42 ^c^ ± 0.005	0.29 ^a^ ± 0.005	***
Gumminess (N)						
30	27.3 ^e^ ± 0.007	17.5 ^b^ ± 0.002	13.93 ^a^ ± 0.005	26.83 ^d^ ± 0.004	18.21 ^c^ ± 0.004	***
60	16.26 ^e^ ± 0.041	11.31 ^c^ ± 0.003	12.59 ^d^ ± 0.001	6.95 ^a^ ± 0.004	10.38 ^b^ ± 0.005	***
90	24.68 ^e^ ± 0.006	10.5 ^c^ ± 0.005	8.81 ^b^ ± 0.002	14.26 ^d^ ± 0.005	5.28 ^a^ ± 0.004	***
Chewiness (N)						
30	13.11 ^e^ ± 0.004	9.28 ^b^ ± 0.001	4.88 ^a^ ± 0.002	11.54 ^d^ ± 0.002	10.20 ^c^ ± 0.003	***
60	5.91 ^e^ ± 0.094	3.47 ^b^ ± 0.093	6.55 ^e^ ± 0.001	2.23 ^a^ ± 0.044	4.69 ^c^ ± 0.043	***
90	10.18 ^e^ ± 0.103	4.52 ^c^ ± 0.002	3.64 ^b^ ± 0.038	5.88 ^d^ ± 0.059	1.65 ^a^ ± 0.022	***

S0 = control—NaNO_2_ 100 mg/Kg; S1 = Hemp seed oil 2%—NaNO_2_ 50 mg/Kg; S2 = Hemp seed oil 4%—NaNO_2_ 50 mg/Kg; S3 = Hemp seed oil 2%; S4 = Hemp seed oil 4%. *** = *p* < 0.001; Superscripts a, b, c, d, e differ at *p* < 0.05.

## Data Availability

The original contributions presented in the study are included in the article, further inquiries can be directed to the corresponding author.
